# Prioritizing Delivery of Cancer Treatment During a COVID-19 Lockdown: The Experience of a Clinical Oncology Service in India

**DOI:** 10.1200/GO.20.00433

**Published:** 2021-01-15

**Authors:** Indranil Mallick, Santam Chakraborty, Shweta Baral, Saheli Saha, Vishnu H. Lal, Rohit Sasidharan, Ritesh J. M. Santosham, Samarth Chhatbar, Subecha Bhusal, Love Goyal, Shaurav Maulik, Vezokhoto Phesao, Siddharth Arora, Tapesh Bhattacharyya, Anurupa Mahata, Sriram Prasath, Arun Balakrishnan, Samar Mandal, Moses A. Arunsingh, Rimpa Achari, Sanjoy Chatterjee

**Affiliations:** ^1^ Department of Radiation Oncology, Tata Medical Center, Kolkata, India

## Abstract

**PURPOSE:**

A COVID-19 lockdown in India posed significant challenges to the continuation of radiotherapy (RT) and systemic therapy services. Although several COVID-19 service guidelines have been promulgated, implementation data are yet unavailable. We performed a comprehensive audit of the implementation of services in a clinical oncology department.

**METHODS:**

A departmental protocol of priority-based treatment guidance was developed, and a departmental staff rotation policy was implemented. Data were collected for the period of lockdown on outpatient visits, starting, and delivery of RT and systemic therapy. Adherence to protocol was audited, and factors affecting change from pre-COVID standards analyzed by multivariate logistic regression.

**RESULTS:**

Outpatient consults dropped by 58%. Planned RT starts were implemented in 90%, 100%, 92%, 90%, and 75% of priority level 1-5 patients. Although 17% had a deferred start, the median time to start of adjuvant RT and overall treatment times were maintained. Concurrent chemotherapy was administered in 89% of those eligible. Systemic therapy was administered to 84.5% of planned patients. However, 33% and 57% of curative and palliative patients had modifications in cycle duration or deferrals. The patient’s inability to come was the most common reason for RT or ST deviation. Factors independently associated with a change from pre-COVID practice was priority-level allocation for RT and age and palliative intent for systemic therapy.

**CONCLUSION:**

Despite significant access limitations, a planned priority-based system of delivery of treatment could be implemented.

## INTRODUCTION

In the wake of the COVID-19 pandemic, India went into a strict lockdown on March 24, 2020. This included the abrupt cessation of all types of public and private transportation except for defined essential services.^1^ The sudden lockdown resulted in oncology services in India facing a decision-making and delivery-of-care crisis.

**CONTEXT**

**Key Objective**
To determine if a priority-based radiation and systemic therapy protocol could be followed during major travel restrictions during a prolonged COVID-19 lockdown.
**Knowledge Generated**
It was possible to start more than 90% of high-priority cancer treatments as scheduled. Ongoing treatments for curative high-priority situations could be continued as planned without additional breaks or lack of compliance.
**Relevance**
Priority-based treatments can be delivered in a planned and systematic manner in a low- or middle-income country despite pandemic restrictions.

Since March 2020, guidelines on cancer treatment and risk-stratified care had started emerging.^2^ These generally suggested modification or deferment of treatment, if considered safe. Although oncology services around the country started adopting one or more of the recommendations,^3-5^ the available literature is limited to consensus guidelines and surveys primarily based on western health-care infrastructure. Implementation of a planned approach from a system that does not have structured state funding for travel and treatment has not yet been audited or reported.

We put in place a detailed protocol to prioritize care pathways using available evidence, biological rationale, and published consensus statements (Data Supplement). We present here an audit of our services from March 24 through May 16, 2020, corresponding to the first to third phases of the lockdown, which posed considerable restrictions on public transport. The focus of this audit was the implementation of treatment delivery among our patients.

## METHODS

### Departmental Triaging and Treatment Protocols

The departmental policy (Data Supplement) was based on the treatment priorities influenced by treatment intent and disease biology. In the absence of any national guidelines by the health ministry or the National Cancer Grid, we devised our own departmental guidelines on March 21, 2020 (just prior to the lockdown), for prioritization of radiotherapy (RT) based on the recommendations of the National Health Service (NHS) UK and divided cancer cases into five levels.^2^ In brief, priority level 1 constitutes rapidly proliferating tumors planned for or undergoing treatment where treatment gaps cannot be effectively compensated or delay in treatment is detrimental. Priority level 2 is malignant spinal cord compression with useful salvageable neurological function. Priority level 3 constitutes less rapidly proliferating tumors where either RT is the first definitive treatment or adjuvant treatment is indicated in known residual disease postoperatively. Priority level 4 is palliative RT for symptoms that would otherwise burden other healthcare services. Priority level 5 is adjuvant RT after complete resection of disease, and there is a < 20% risk of recurrence at 10 years or radical RT for prostate cancer in patients receiving neoadjuvant hormone therapy.

For patients on systemic therapy, we did not use the NHS guideline but used a simpler priority system based on the curative versus palliative intent of therapy. We maintained a prospective database of all patient cases where treatment was deferred during this period.

### Data Sources

We obtained patient visit data between January 1 and May 16 for the years 2019 and 2020 from the electronic hospital information system (HIS). The HIS and oncology information system, ARIA, (Varian Medical Systems, Palo Alto, CA) were queried to obtain information on patient characteristics and treatment delivery patterns between March 24 and May 16, 2020. As a comparative data set, RT bookings and deliveries for the corresponding period in 2019 were audited focusing on nonstarts and delays in patients with priority levels 1-3. Study data were collected and managed using REDCap electronic data capture tools.^6,7^ The audit received a waiver of consent and detailed review from the institutional review board (EC/WV/TMC/33/20).

### Statistical Analysis

R^8^ and Python 3 were used for statistical analysis. The χ^2^ test and the Kruskal-Wallis test were used for statistical testing of differences in frequencies and continuous variables, respectively.

Multivariate modeling was used to identify the factors predicting deviation of RT and chemotherapy from pre-COVID protocols (Data Supplement). For RT, the protocol deviations were defined as any of the following: RT indicated but not started or started with deferral; priority level 1 patients starting RT more than 6 weeks after surgery/last day of neoadjuvant or adjuvant chemotherapy; and patients with overall treatment time (OTT) > 3 days over the planned duration of RT or planned treatment not completed. For chemotherapy, the protocol deviations considered were chemotherapy deferral, any change in chemotherapy schedule in terms of drug dose or interval modifications, or incomplete chemotherapy. Multivariate analysis was performed using logistic regression, where the presence of any deviation was considered as the independent variable. Model predictors were added linearly, and no interactions were assumed. Odds ratios (ORs), 95% CIs, and *P* values are presented. A *P* value of < .05 is considered statistically significant.

## RESULTS

### Outpatient Visits

**FIG 1:**
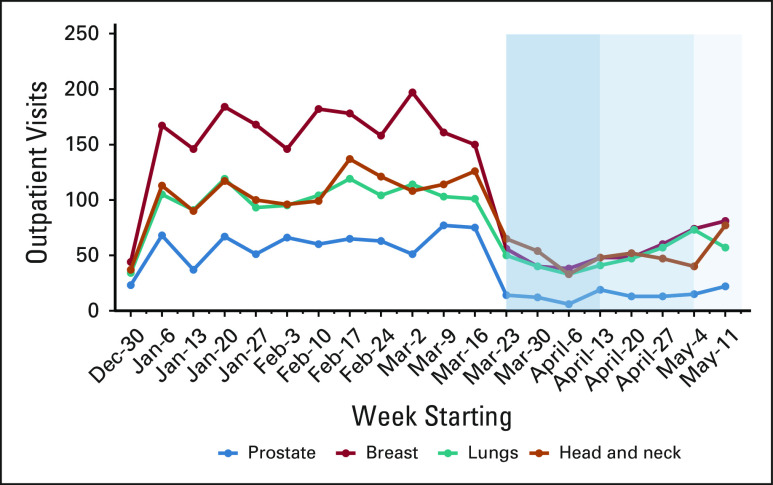
The change in case load in the outpatient clinics in the period corresponding to the first, second, and third phases of the lockdown (weeks 13-15, 16-18, and 19-20) for major cancer site groups.

Between January 1 and May 16, a total of 5,291 and 5,090 patients had outpatient visits in 2019 and 2020, respectively. There were 12,325 outpatient consultations between January 1 and May 16, 2020, compared with 13,140 in the same period in 2019. Although there were 1,983 (25%) excess outpatient visits in the first 12 weeks of 2020 as compared to 2019, there was a sharp decline in patient visits induced by the lockdown in the 13th week. The average weekly follow-up visits in the four most common groups of cancers (breast, lung, head and neck, and prostate) dropped by 65%, 49%, 50%, and 76%, respectively (Fig 1).

### Radiation Therapy

During the lockdown period, there were 305 patients who were planned to start RT from March 24 to May 15, 2020. Of these, 262 patients were able to start the treatment by May 31, 2020. The compliance and reasons for noncompliance or delays are presented in Table 1. Breast (27%), head and neck (23%), and lungs (17%) were the most common sites (Data Supplement). Of the 145 patients in priority levels 1, 2, and 3, a total of 132 (91.0%) could begin their treatment during the lockdown.

**TABLE 1:**
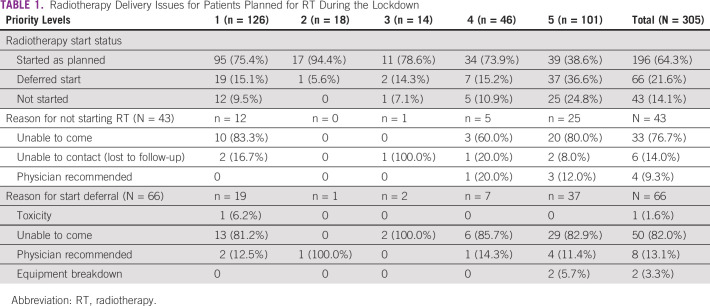
Radiotherapy Delivery Issues for Patients Planned for RT During the Lockdown

In 125 patients (47.7%), either adjuvant RT after surgery or definitive RT after induction chemotherapy was performed. Of these, 54 patients had priority level 1, whereas 69 had priority level 5. The median time to start RT was 40 days (range, 14-69 days) after surgery or the last cycle of chemotherapy. A delay of 6 weeks in starting RT was observed in 17 (31.5%) patients. In all but one of these patients, the delay beyond 6 weeks was due to restrictions in patient travel, finances, or delayed attendance in our hospital after a surgery done in another hospital. Among priority level 5 patients undergoing adjuvant RT, the median gap between RT and the last cycle of chemotherapy or surgery was 53 days (range, 16-110 days).

The median RT plan turnaround time (TAT) was 7 days (range, 0-44 days). Among priority level 1 patients, plan TAT exceeding 14 days was observed in 3 (2.6%) patients. Plan TAT did not exceed 14 days in any of the priority 1 patients on adjuvant or postinduction RT. All patients in priority level 2 started on the same day of planning.

During the same time period in 2019, 225 patients were planned for RT start. A total of 105 of these patients would be considered as priority levels 1-3. Eighty-one patients (77.1%) started treatment: 70 patients started on time, and 11 had a start delayed by more than 1 day. Of the remaining 24 patients, 12 (11.5%) did not report for RT planning, five more did not start because of worsening performance status, and five patients went to other hospitals for an earlier treatment start. Of the 11 delayed patients, with a median delay of 5 days, the common reasons were postsurgical or postchemotherapy toxicity and patients inability to come for treatment start on time. For priority level 1 patients starting adjuvant therapy, the median time between surgery and RT was 39 days, and 2 of the 15 patients exceeded 6 weeks primarily as a result of postsurgical morbidity.

### Concurrent Chemotherapy.

Concurrent chemotherapy was indicated in 65 of the 203 patients (32.0%), of whom 58 patients (89.2%) were started on chemotherapy.

In seven patients where planned concurrent chemotherapy was not started, COVID-related concerns predominated, with age ≥ 70 and borderline performance status. One patient had a squamous cell carcinoma of the skin (in which evidence of concurrent chemotherapy was felt to be less robust). Two patients were planned to be started on chemotherapy but found to have multiple comorbidities and poor tolerance early into treatment.

Concurrent chemotherapy could not be delivered per protocol in 16 of 58 patients (27%) because of treatment-related toxicities, with a lower number of concurrent cycles than planned because of skipped cycles or an earlier termination.

In comparison, in 2019, 33 patients were planned for concurrent chemotherapy. All were started on chemotherapy. Chemotherapy could not be delivered per protocol in six patients (18.2%), again primarily because of early stoppage due to toxicity.

### Delivery of RT.

Four hundred and thirty patients underwent RT during the lockdown (262 new and 168 ongoing) (Table 2, Data Supplement). By deferring starts of priority level 4 patients, on-treatment numbers reduced to an average of 129 per day during the lockdown period from 172 earlier (Fig 1, Data Supplement). A total of eight patients had breaks, and six patients could not complete their planned treatment. Of the six, two were unable to come for further therapy, whereas the remaining progressed or died during the treatment (unrelated to COVID). OTT was prolonged by more than 3 days in 14 patients (five priority level 1 patients). There was no significant difference in the radiation delivery patterns among the patients who were started during the lockdown period vis-à-vis those who continued treatment during the lockdown (Data Supplement).

**TABLE 2:**
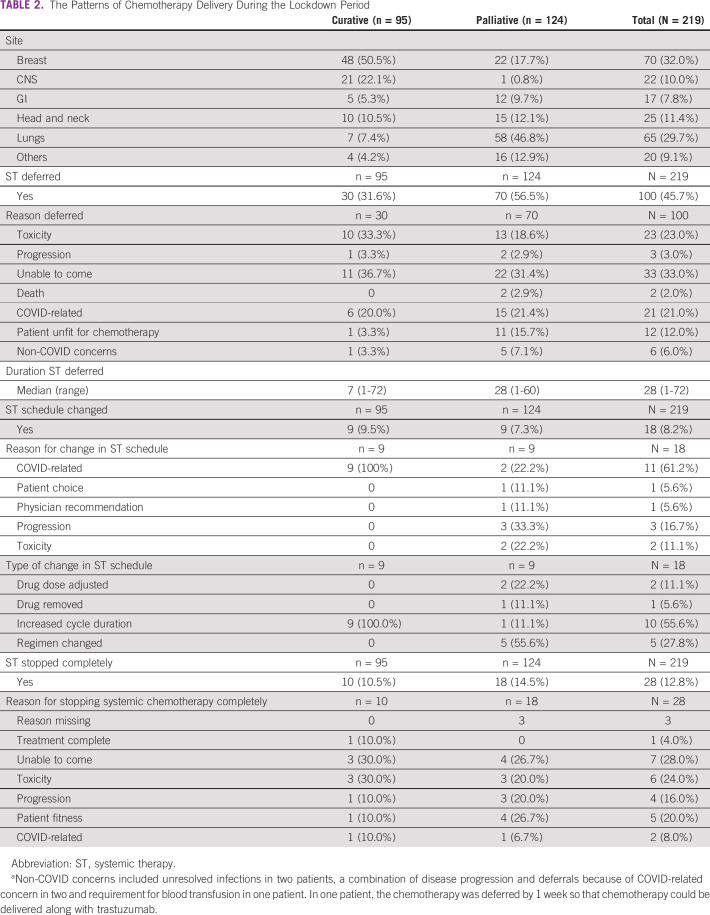
The Patterns of Chemotherapy Delivery During the Lockdown Period

In 2019, none of the 81 patients who received RT had a treatment prolongation of more than 3 days, and none of the priority level 1 patients had any prolongation. One patient’s treatment was curtailed after on-treatment imaging showed disease progression in an inoperable skull base tumor after 5 weeks.

### Downtime.

We faced a great challenge with downtime, with one of the four treatment units down for technical reasons on 24 of the 59 working days (inclusive of Saturdays). However, timely shifting of patients to alternative units was done, which is reflected in the OTT.

### Brachytherapy.

Twenty-five patients with gynecological cancers were planned for brachytherapy. Of these, brachytherapy could be delivered in 17 patients (11 with cervical cancer, five with endometrial cancer, and one with vault recurrence). A scheduled brachytherapy source exchange had to be deferred during the lockdown as a result of which eight patients (four with cervical cancers and endometrial cancers each) were referred outside for brachytherapy after May 1, 2020 (as the unit could not deliver treatment). For three patients, an altered intracavitary brachytherapy dose fractionation schedule was used (9 Gy in two fractions) instead of the usual 7 Gy in three fractions, in anticipation of delay in source exchange.

In the 17 patients who received brachytherapy, treatment was completed in 15 patients. Two patients with endometrial cancers were unable to come for the last fraction of vaginal brachytherapy. The total duration of treatment for patients who underwent treatment during this period was ≤ 56 days for all except one patient.

### Factors Affecting Deviation from Pre-COVID Usual Radiotherapy Practice.

**FIG 2:**
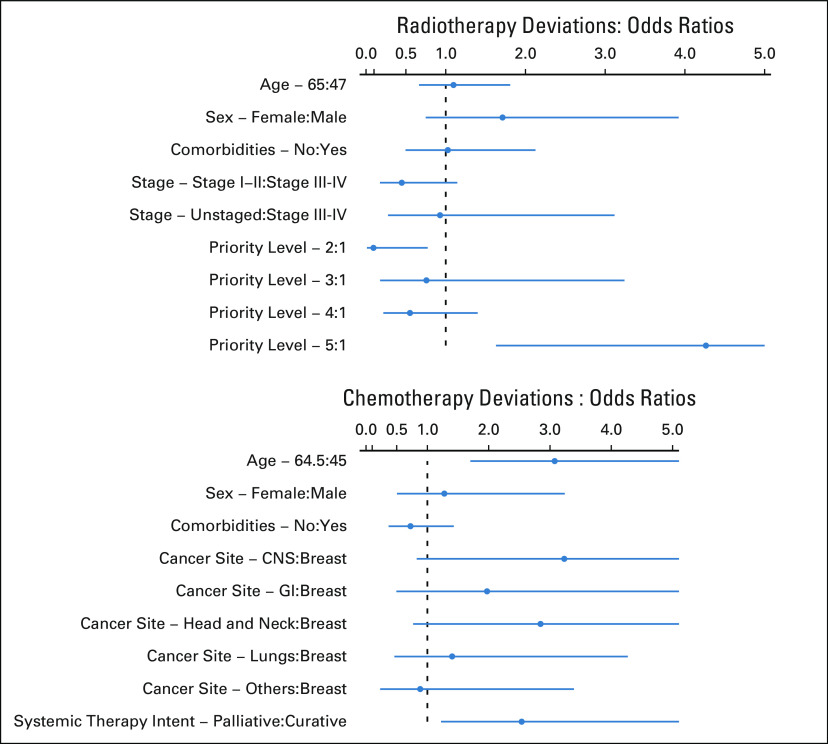
The odds ratio and 95% CIs of the estimate for each variable obtained from logistic regression for radiotherapy (panel A) and chemotherapy (panel B) protocol deviations. The indicator value is toward the right of the colon sign. The x-axis of the plot is trimmed at 5.0. Age was modeled as a continuous factor, and therefore, interquartile range effects have been presented.

Factors affecting deviation from practice are shown in the Data Supplement. Figure 2 (panel A), online only shows that the only factor that was independently associated with deviation from pre-COVID protocol was the priority level. Compared with priority level 1, priority level 5 had an OR of 4.02 (1.53-10.63, *P* = .005) for a change or deviation. Priority level 2 had less deferment—an OR of 0.08 (0.01-0.71, *P* = .02). This was in accordance with our protocol during the lockdown.

## Systemic Therapy

### Starting Planned Systemic Therapy.

Systemic therapy was indicated in 395 patients, of whom 61 patients could not start the treatment during the lockdown period. The most common reasons for this were patient default (n = 31, 50.8%), patient unfitness to receive systemic therapy (n = 13, 21.3%), and COVID-19–related concerns (n = 8, 13.1%).

### Compliance in Those Who Received Chemotherapy.

After excluding patients for targeted therapy (oral tyrosine kinase inhibitors, immunotherapy, and monoclonal antibodies), chemotherapy was delivered in 219 patients. Ninety-five patients (43.4%) received curative-intent treatment. Combination chemotherapy was used in 126 patients (57.5%).

Table 2 shows the implementation of chemotherapy in these patients. About one-third of curative-intent chemotherapy and close to 60% of patients on palliative chemotherapy had some form of deferral from planned dates during the lockdown. The median duration of delay was longer in palliative patients (28 days *v* 7.5 days, *P* = .002). Deferrals in curative patients were equally related to disease or toxicity-related causes, inability to attend because of the lockdown, and physician recommendations. Deferrals in patients on palliative systemic therapy were more commonly due to physician recommendation (56.4%). In a smaller proportion of patients, there was a change in chemotherapy schedule, mainly related to reduced intensity. In only 26 patients (12%), chemotherapy was stopped completely, and 18 of these patients were on palliative treatment. Toxicity-related stoppage or deferral was relatively uncommon.

### Factors Affecting Deviation from Pre-COVID Chemotherapy Practice.

Some form of deviation from prechemotherapy practice (as defined earlier) was observed in 120 of the 219 patients (54.7%, Data Supplement). Factors affecting deviation from usual practice are shown in the Data Supplement. Figure 2 (panel B) shows that the two factors independently associated with deviation from pre-COVID chemotherapy practice were increasing age (OR between third and first quartiles 3.48, 95% CI, 1.71-7.07, *P* ≤ 0.01) and palliative-intent chemotherapy (OR, 3.03, 95% CI, 1.28-7.14, *P* = .01), which reflects our modified intent during the lockdown.

## DISCUSSION

When the lockdown was imposed nationwide in India on March 24, the state of West Bengal had nine confirmed cases and one death because of COVID-19, increasing to 2,532 cases and 232 deaths on May 16, 2020.^9^ While this reduced casualties from COVID-19, the lockdown had wide-ranging effects on other healthcare services.

The magnitude of the effect of the COVID-19–induced lockdown on cancer care is emerging globally.^10,11^ Data from Prime Minister Jeevandayee Arogya Yojana, a universal health insurance scheme, show claims related to oncological care that fell by nearly 64% during the lockdown.^12^ Systemic chemotherapy deferrals and delayed start of new patients on chemotherapy were responsible for this decline. Similar reports of disruption in oncological care delivery and its impact have emerged from other healthcare delivery systems in Germany,^13^ Japan,^14^ Italy,^15,16^ and the United Kingdom.^17,18^

To ensure service continuity, services have adopted a system of staff rotations during this crisis.^4,16,19,20^ Treatment prioritization enabled us to continue delivering safe treatment with reduced staff. Our team was able to reach out to patients with scheduled appointments and provide guidance based on priority levels. This is reflected in a greater drop in follow-up patient visits in patients with breast and prostate cancer. As can be appreciated from the outpatient visit data, although the clinic visits in January and February 2020 were considerably higher than those in 2019 as a result of a hospital expansion, there was a sharp postlockdown drop in clinic attendance. This was a combined effect of lack of transport and telephonic contact with patients deferring routine follow-up visits.

In terms of RT services, we took the decision of not postponing or interrupting RT in patients who were already undergoing treatment. In hindsight, this decision was proven correct as, to date, there is no sign that the epidemic is abating in India despite the lockdown. However, by deferring new starts for priority level 5 patients, we were able to reduce the new starts. This ensured that manpower for planning and treatment could be strategically redeployed by rotation based 50% attendance of radiation therapists and medical physicists or dosimetrists to prevent delays and ensure safety in delivering full services for priority level 1-3 and symptomatic level 4 patients.

For priority level 1-3 patients, this strategy succeeded in implementing more than 90% of planned starts. Deferments and incomplete treatments were linked primarily to patients being unable to come for treatment. Concurrent chemotherapy was also successfully implemented in the majority. The only factor predicting a change from pre-COVID RT practice was the priority level assigned, which matched with our intent.

In comparison with 2019, we were no worse in 2020 in terms of RT start and concurrent chemotherapy. Approximately 10% of patients did not come back for their planned treatment start in either year. It must be noted that in 2019, we had only two linear accelerators instead of four in 2020, and therefore, the numbers booked were lower; we had a longer waiting list, prompting some patients to go to other hospitals. In view of the difference in circumstances, a head-to-head comparison of dropout rates would not be ideal.

The department continued to offer specialized procedures that are highlighted by the fact that one patient successfully underwent total body radiation as a part of the conditioning regimen for bone marrow transplant.^21^ Additionally, complex planning techniques were used as indicated and no change in the planning technique was made. For example, all patients with breast cancer continued to be treated with cardiac sparing using a deep inspiration breath hold (using a Varian Real-time Position Management^®^ system).^22^ No changes in anesthesia procedures were made. All brachytherapy insertions were performed under anesthesia (general anesthesia or regional). This is unlike the experience in some western centers, where similar complex procedures were suspended.^23^ Significant changes in our dose fractionation schedules were not required as our pre-existing departmental policy was to use hypofractionated RT wherever it was safe.^24-26^

The two factors that predicted a deviation from the usual pre-COVID chemotherapy delivery were age and use of palliative chemotherapy—both of which were in line with our proposed departmental protocol (Data Supplement). For curative-intent patients, however, a change in dose density was offered. This is reflected in the duration of the deferral of chemotherapy. We also offered G-CSF-based prophylaxis to all patients.

Similar experiences from other centers are yet to be reported. No episodes of transmission of COVID-19 from staff to patient or vice versa were observed on symptom-based testing. Daily pretreatment screening and appropriate counseling of patients and staff may have contributed. However, during this period, there was no systematic testing of patients taken up for treatment. Government regulations only allowed symptom-based tests. Elective testing before RT and OT was adopted after the government approved the policy of walk-in testing for patients on 26 May 2020 (vide circular HPH/9M-21/2020/110^27^). Similarly, we followed government advisory for staff testing, which was only done for symptomatic staff members. Furthermore, with the support of the administration, we were able to ensure that staff could travel from far-flung areas in the city. We must acknowledge the fact that the patients understood the importance of their disease and its treatment and were motivated to continue on the treatment during this period.^28^

This audit has several limitations. We were unable to audit the compliance to targeted therapy, oral tyrosine kinase inhibitors, or immunotherapy, as it would have considerably increased the requirement of manual review of electronic medical records prior to the lockdown, and required detailed telephonic contact with patients for which we did not have sufficient manpower. For the same reasons, we could not compare the 2019 experience for adjuvant and palliative systemic therapy. We also did not statistically compare treatment compliance between the period of audit and the corresponding period in 2019 as a large hospital expansion in June 2019 changed several baseline circumstances, including patient waiting lists.

In conclusion, the audit demonstrates that during significant restrictions of the countrywide lockdown for the COVID-19 pandemic, a planned priority level–based approach toward cancer treatment could be implemented and allow cancer care to be delivered to the patients most in need of early treatment, while reducing risk and hospital visits for patients with cancers that allowed for planned deferment, the elderly, and those treated with palliative intent. However, in terms of delayed diagnoses or delayed access for newly diagnosed patients in the community, the other downstream effects of the lockdown will only become apparent with further follow-up. Modeling results suggest a significantly increased risk of death due to delayed treatment and diagnosis in this population.^29^

## PREPRINT VERSION

Preprint version available on Researchsquare.com (https://dx.doi.org/10.21203/rs.3.rs-38180/v1).
